# Challenges in Measuring What Matters to Patients With Diabetes. Comment on “Measurement Properties of Patient-Reported Outcome Measures for Diabetes: Systematic Review”

**DOI:** 10.2196/36876

**Published:** 2022-03-31

**Authors:** Femke Rutters, Ellen Elsman, Lenka Groeneveld, Marlous Langendoen-Gort, Lidwine Mokkink, Caroline Terwee

**Affiliations:** 1 Department of Epidemiology and Data Science Amsterdam University Medical Centre, Location Vrije Universiteit Amsterdam Amsterdam Netherlands

**Keywords:** systematic review, measurement properties, patient-reported outcome measures, methodological quality, level of evidence, PROMs, patient reported outcome, diabetes

We would like to respond to Wee et al’s paper, “Measurement Properties of Patient-Reported Outcome Measures for Diabetes: Systematic Review” [1]. We appreciate the herculean effort undertaken to summarize all diabetes-related patient-reported outcome measures (PROMs). However, we have some concerns.

First, despite the large amount of identified PROMs (N=238), there are still many PROMs missing [1]. In our systematic review of PROMs measuring health-related quality of life (HRQOL) in people with type 2 diabetes (currently under review), which was performed in the same time period and using the same databases, we identified 116 HRQOL PROMS. Of these, >50 were missing in Wee et al’s review [1]. Missing PROMs include, for example, the National Diabetes Register Survey [2], which in our review showed the best content validity. We think this incompleteness is due to a lack of alternative search strategies, such as checking references. We were surprised that the authors [1] identified no papers through hand-searching, while about one-fourth of the included papers in our review were identified through reference checking.

Second, the authors [1] used the COSMIN (Consensus-Based Standards for the Selection of Health Measurement Instruments) methodology to summarize the evidence on the quality (measurement properties) of the PROMs. However, contrary to the COSMIN guidelines, the quality of the PROMs was not rated for each PROM subscale separately, even though measurement properties can vary among subscales.

The limitations of this review [1] underscore the problematic status of PROMs in diabetes: there is no consensus on what doctors and scientists want to measure, and it is unclear what is most relevant to measure. The content of the existing PROMs is very heterogeneous; there are too many PROMs out there and many are of questionable validity. This hinders value-based health care and limits the value of PROMs when attempting to determine which treatment works most optimally. More awareness is needed, supported by recent initiatives on developing core outcome sets for people with diabetes [3-5]. We should start using those core outcome sets in our research and care for people with diabetes.

In conclusion, there is still a need for a high-quality systematic overview of all available PROMs for people with diabetes, with emphasis on the constructs being measured, and a comprehensive evidence synthesis of the measurement properties of all (subscales of) PROMs. This would allow researchers and doctors working with people with diabetes to make informed choices on which PROMs to use for value-based health care.

## Abbreviations

COSMIN: Consensus-Based Standards for the Selection of Health Measurement Instruments
HRQOL: health-related quality of life
PROM: patient-reported outcome measure
